# Supplementary material for: Operando magnetic resonance imaging for mapping of temperature and redox species in thermo-electrochemical cells
Source: Nat Commun. 2021 Nov 8;12:6438. doi: 10.1038/s41467-021-26813-8 (PMC8575911; doi:10.1038/s41467-021-26813-8)
Supplement: Supplementary file 1 — Supplementary Information [file 41467_2021_26813_MOESM1_ESM.pdf]

Operando magnetic resonance imaging for mapping of temperature and redox species in thermo-electrochemical cells

Supplementary Information

Isuru E. Gunathilaka[a], Jennifer M. Pringle[b] and Luke A. O'Dell[a]*

[a]ARC Centre of Excellence for Electromaterials Science (ACES), Institute for Frontier Materials, Deakin University, Geelong Waurn Ponds Campus, Victoria 3220, Australia

[b]ARC Centre of Excellence for Electromaterials Science (ACES), Institute for Frontier Materials, Deakin University, Melbourne Burwood Campus, Victoria 3125, Australia

*Email: luke.odell@deakin.edu.au

Contents **Page**

Cell drawings	2
Experimental set up schematic	4
^{1}H NMR spectrum of the electrolyte	5
Polynomial function details	6
Additional temperature maps for the vertical electrode cell with the liquid electrolyte	7
1 mm thick slice temperature maps of the electrolyte at various positions/orientations	8
Plots of measured current and voltage as a function of time for all series of images	10
Images and average output power for the vertical electrode cell with the gel electrolyte	15

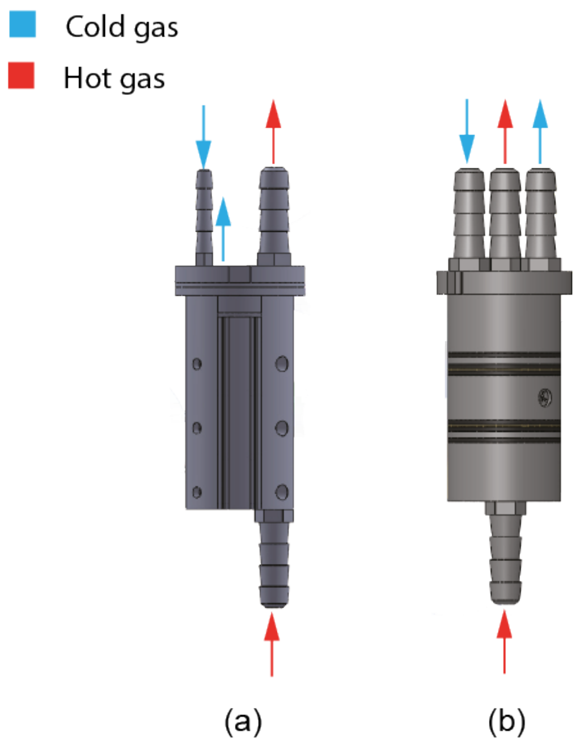

Supplementary Figure 1 – External view of the (a) vertical electrode and (b) horizontal electrode cells.

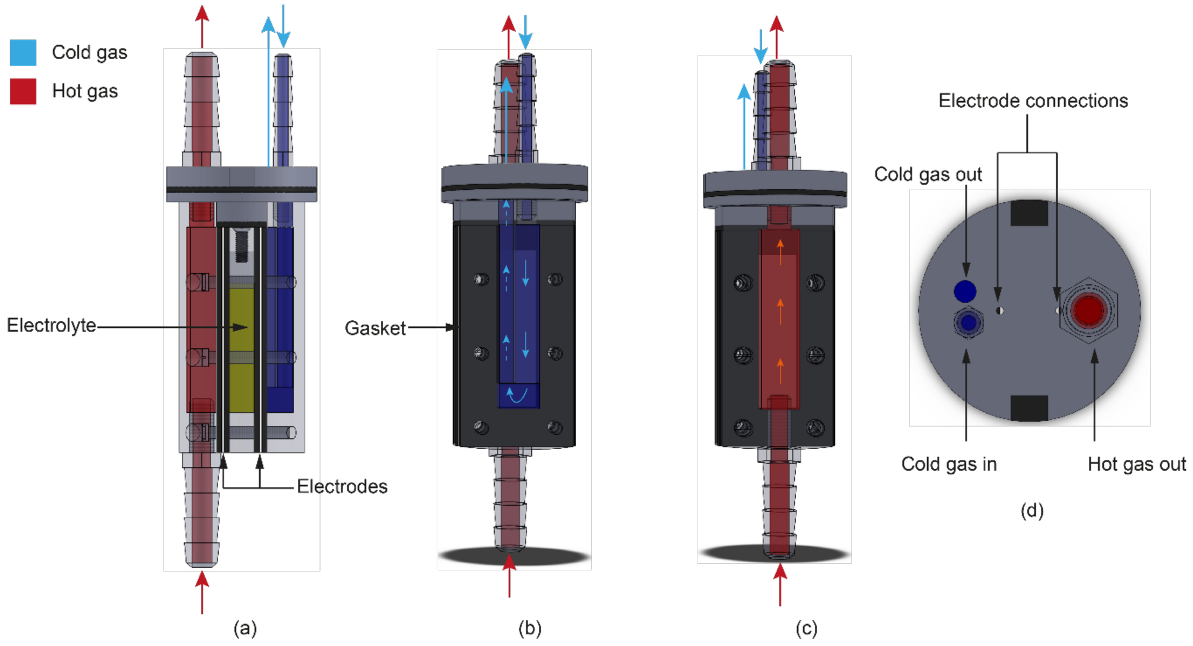

Supplementary Figure 2 – Interior views of the vertical electrode cell showing the (a) electrolyte chamber, (b) the cold gas pathway and (c) the hot gas pathway. (d) Top view of the cell cap.

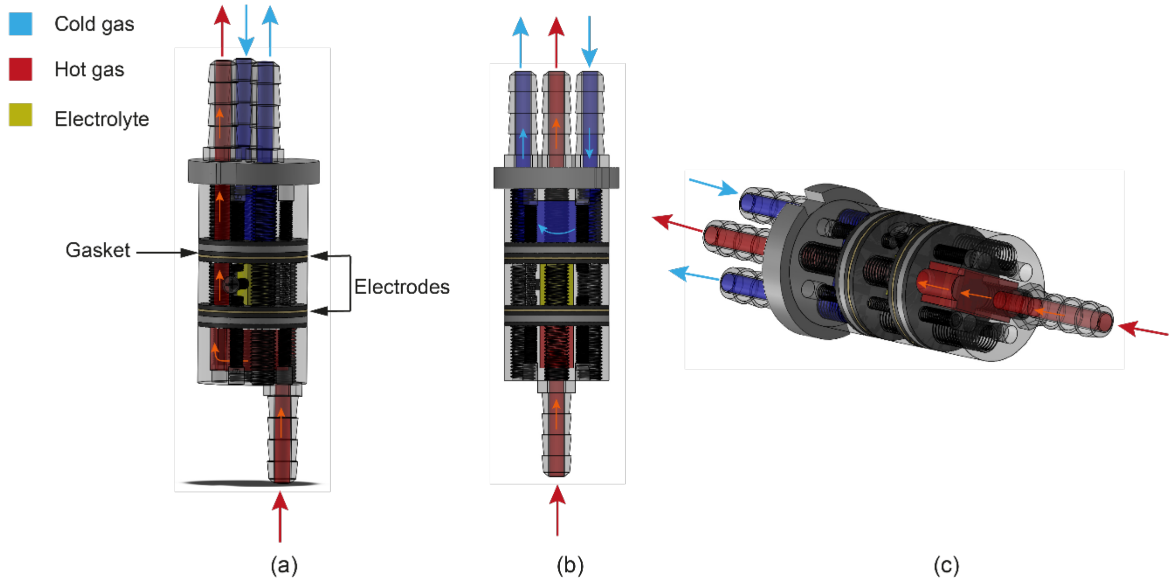

Supplementary Figure 3 – Interior views of the horizontal electrode cell in cold-above-hot configuration, with (a) the hot gas pathway and (b) the cold gas pathway visible. (c) Alternative view.

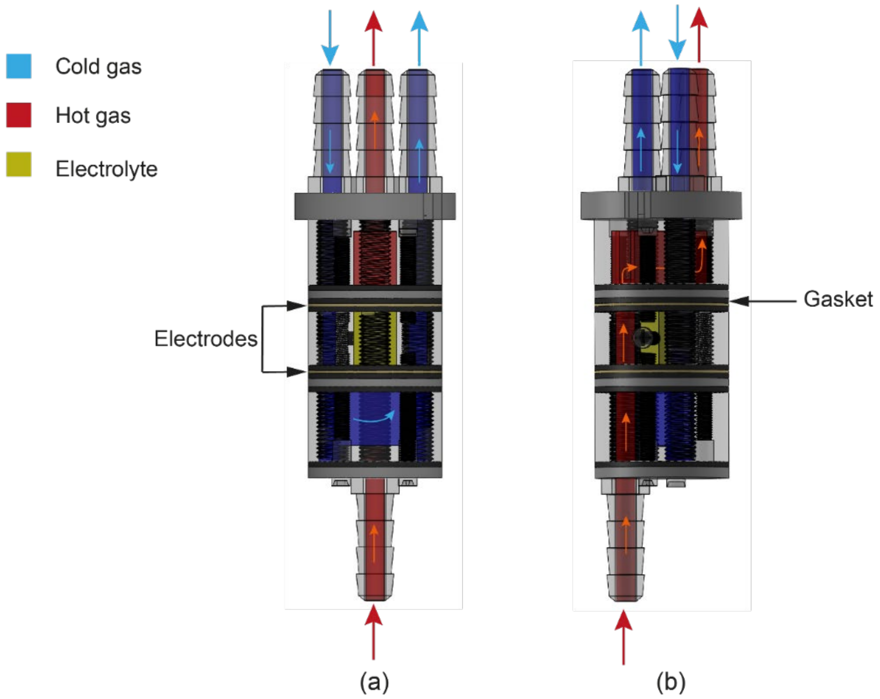

Supplementary Figure 4 – Interior views of the horizontal electrode cell in hot-above-cold configuration, with (a) the cold gas pathway and (b) the hot gas pathway visible.

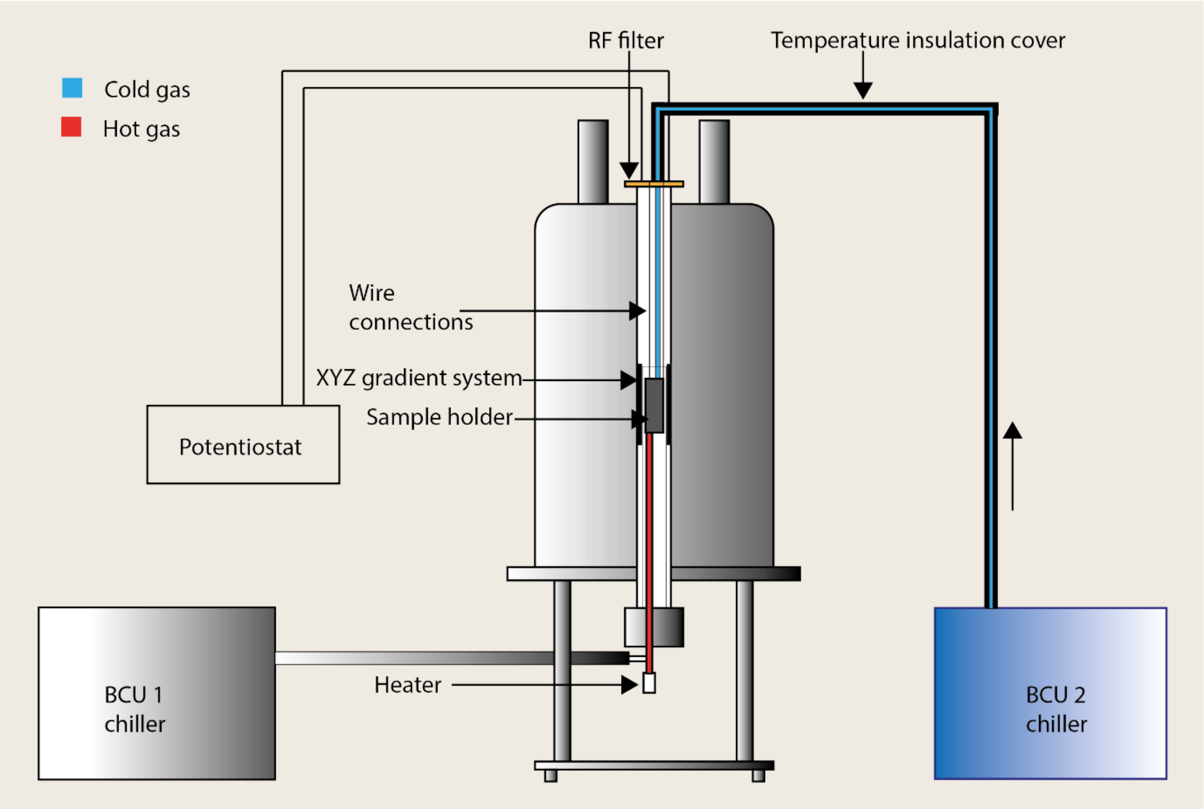

Supplementary Figure 5 – Schematic illustration of the operando MRI experimental set up with connections to potentiostat and chiller units shown.

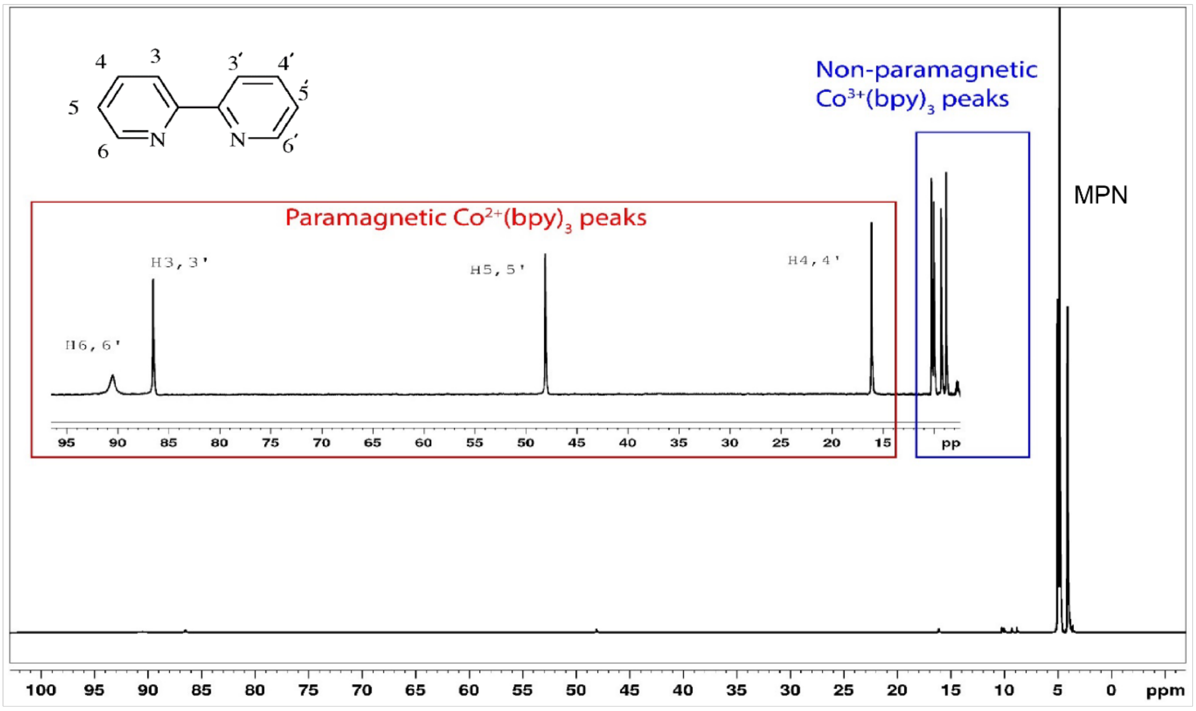

Supplementary Figure 6 – ^{1}H NMR spectrum of the electrolyte (MPN containing 0.05 M $Co^{2+}(bpy)_3(TFSI)_2$ and 0.05 M $Co^{3+}(bpy)_3(TFSI)_3$), with the bipyridyl peaks assigned for the paramagnetic Co^{2+} state.

Function used to fit the ^{1}H T_1 relaxation data in Figure 2f:

$$T_1(C,\theta) = p_{00} + p_{10}C + p_{01}\theta + p_{20}C^2 + p_{11}C\theta + p_{02}\theta^2 + p_{30}C^3 + p_{21}C^2\theta + p_{12}C\theta^2 + p_{03}\theta^3$$

where C is the Co^{2+} concentration and θ is the electrolyte temperature in °C.

Coefficients (with 95% confidence bounds):

$p_{00} = 1448 \ \ (1424, 1472)$

$p_{10} = 353.8 \ \ (317.4, 390.3)$

$p_{20} = -459.8 \ \ (-495.5, -424.2)$

$p_{11} = -4.451 \ \ (-19.01, 10.11)$

$p_{02} = 254.6 \ \ (240.2, 269)$

$p_{30} = -1.832 \ \ (-19.67, 16.01)$

$p_{21} = 11.12 \ \ (-3.521, 25.76)$

$p_{12} = 62.1 \ \ (47.63, 76.57)$

$p_{03} = -110.9 \ \ (-128, -93.86)$

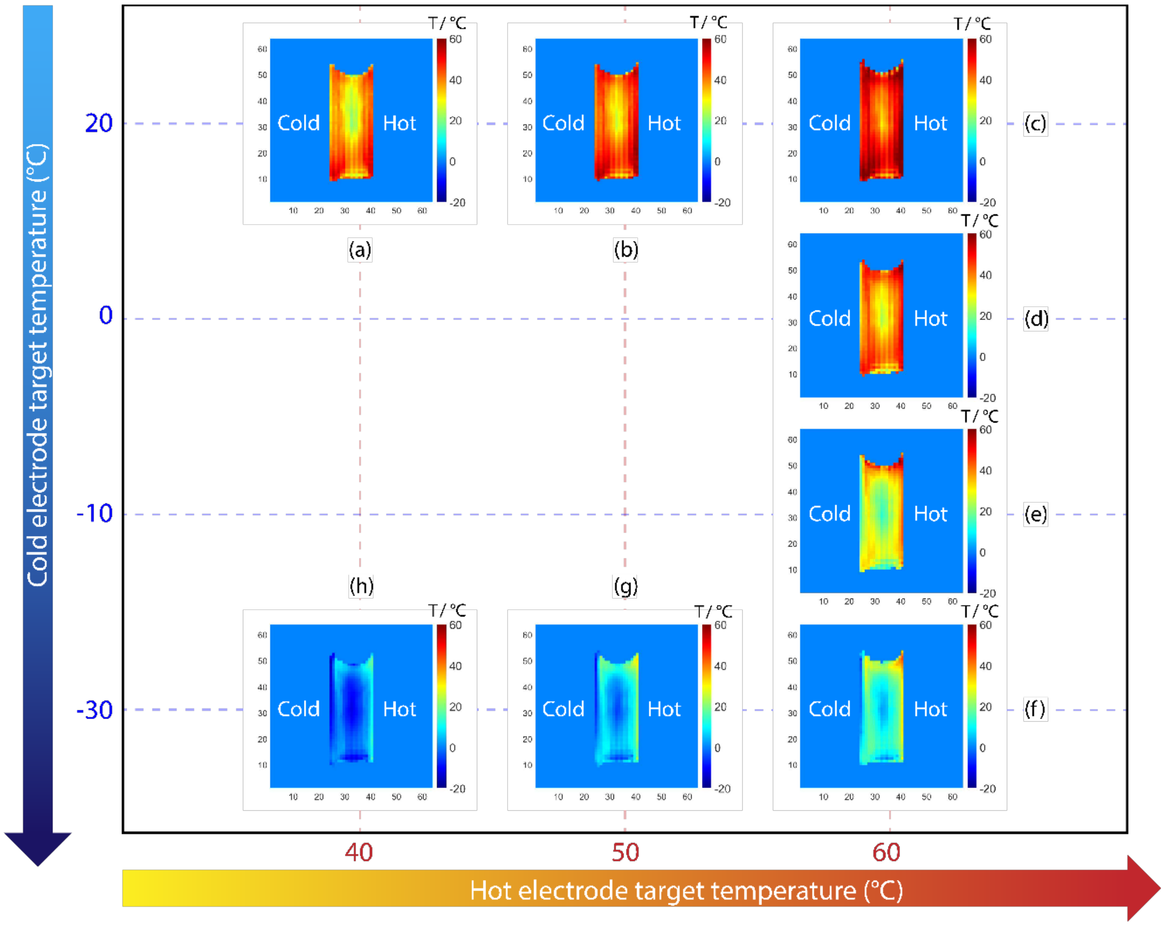

Supplementary Figure 7 - Variation of the temperature distribution in the vertical electrode thermocell with the liquid electrolyte under different applied target temperature differentials.

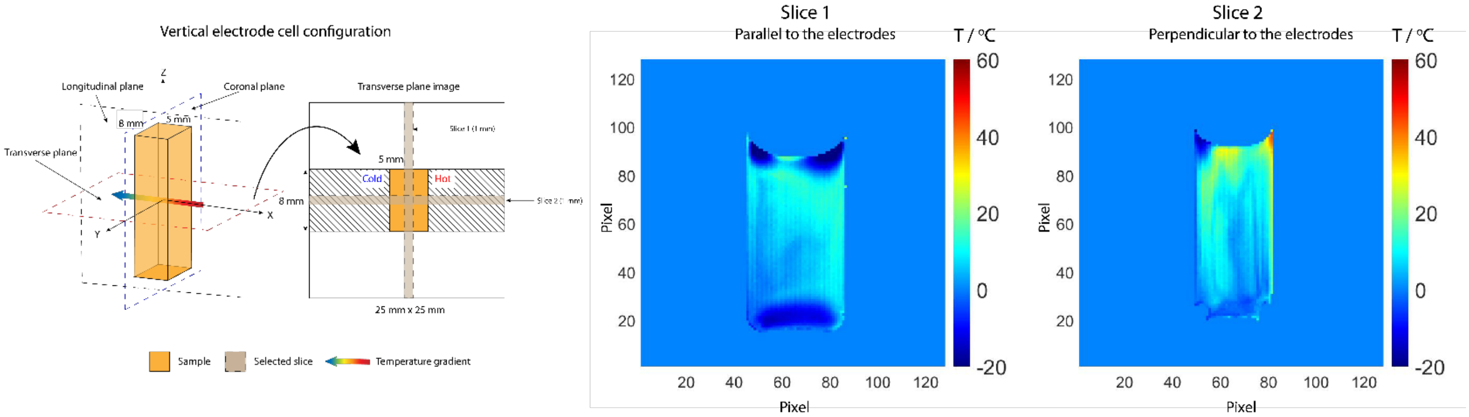

Supplementary Figure 8 – Temperature maps of the vertical electrode thermocell with the liquid electrolyte corresponding to 1 mm thick slices with orientations as indicated. The target temperatures for the hot and cold electrodes were 60 and −20 °C respectively.

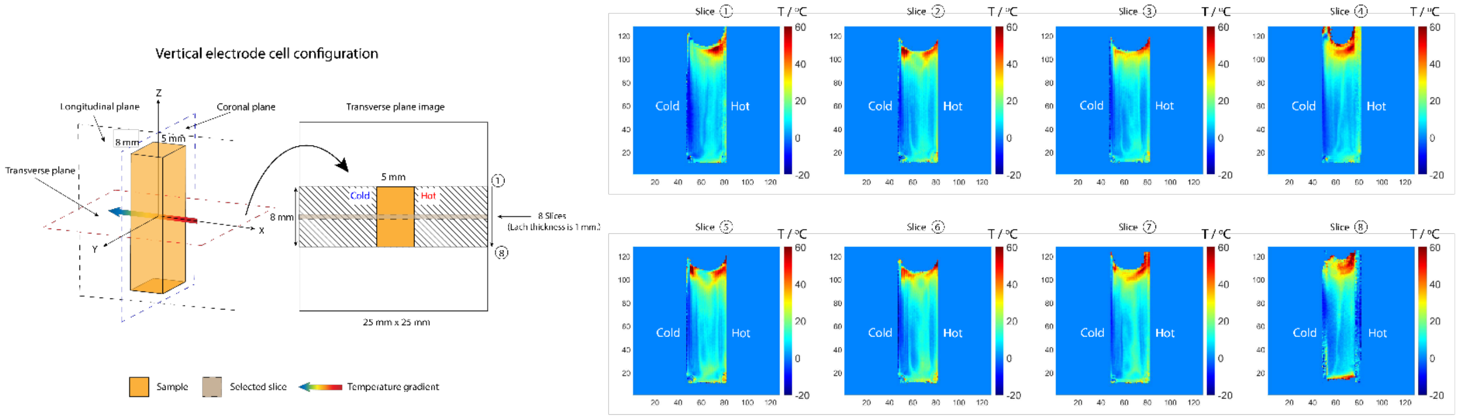

Supplementary Figure 9 – Temperature maps of the vertical electrode thermocell with the liquid electrolyte corresponding to a series of equally-spaced 1 mm thick slices with orientation as indicated. The target temperatures for the hot and cold electrodes were 60 and −20 °C respectively.

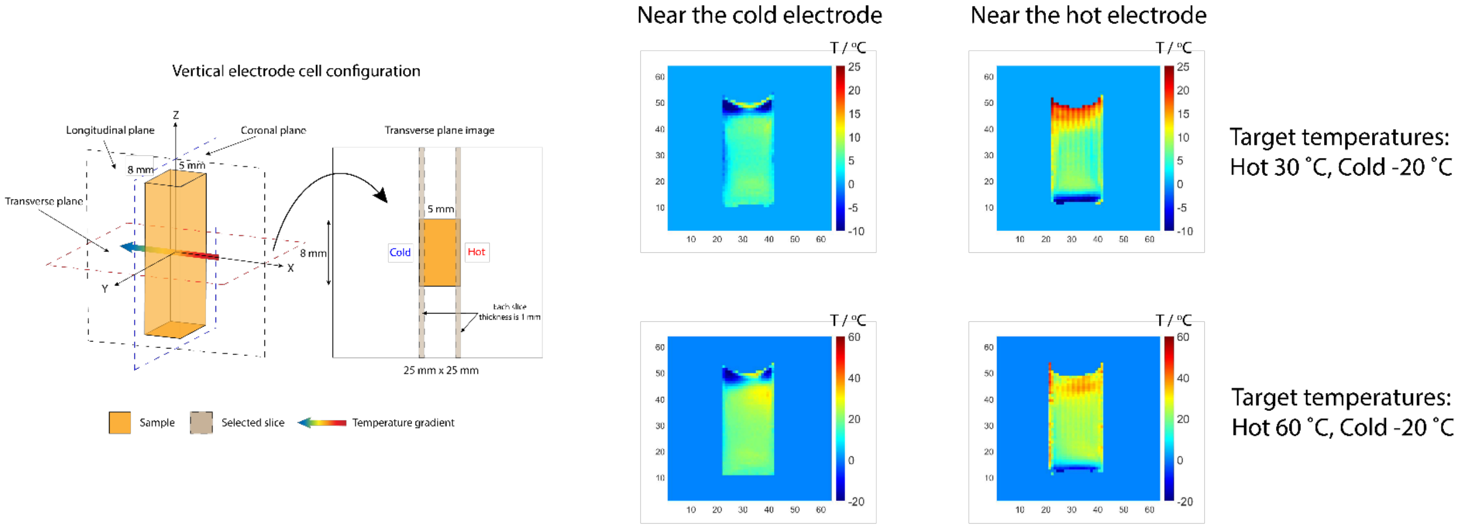

Supplementary Figure 10 – Temperature maps of the vertical electrode thermocell with the liquid electrolyte corresponding to 1 mm thick slices placed close to and parallel to the electrode surfaces as indicated. The target temperatures for the hot and cold electrodes are indicated to the right of the images.

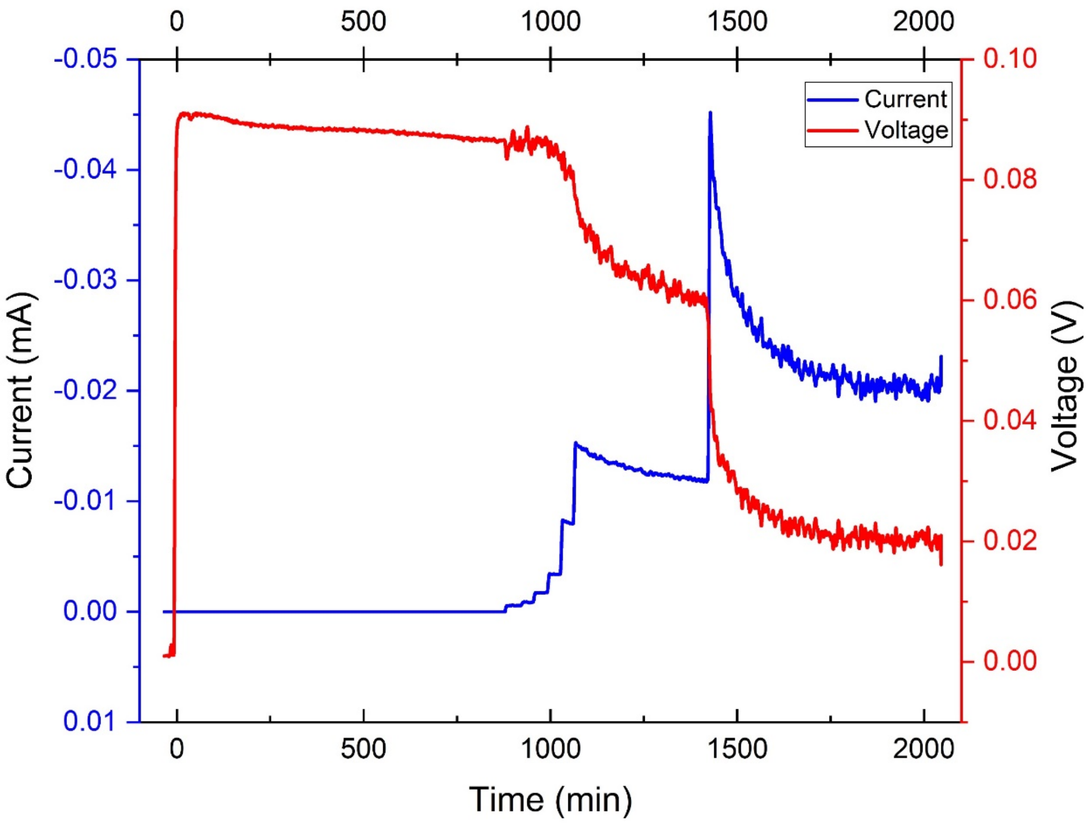

Supplementary Figure 11 - Plot of current and voltage against experimental time with the different applied resistances for the vertical cell with the gel electrolyte images shown in Figure 4. The following external load resistances were connected at the following times: 150 kΩ - 878 min, 100 kΩ - 920 min, 50 kΩ - 953 min, 25 kΩ - 990 min, 10 kΩ - 1026 min, 5 kΩ - 1061 min, 1 kΩ - 1421 min.

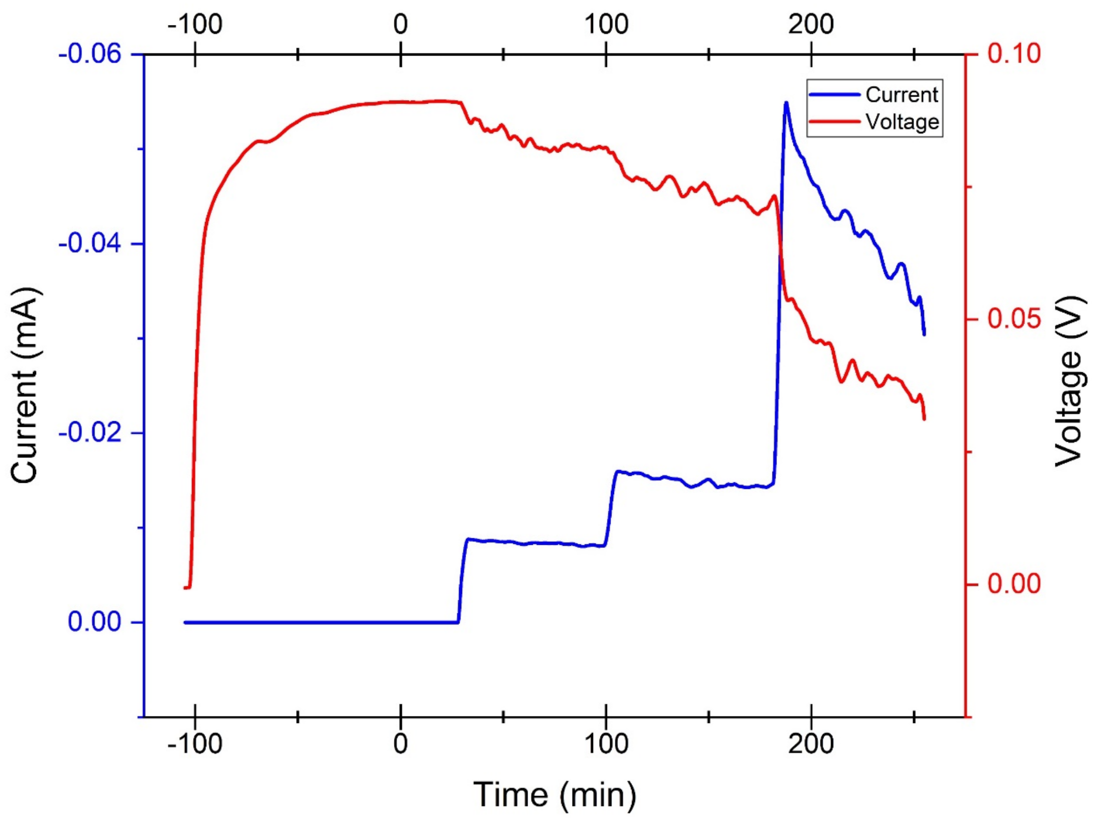

Supplementary Figure 12 - Plot of current and voltage against experimental time with the different applied resistances for the vertical cell with the gel electrolyte images shown in Figure 5. The following external load resistances were connected at the following times: 10 kΩ - 28 min, 5 kΩ - 101 min, 5 kΩ - 183 min.

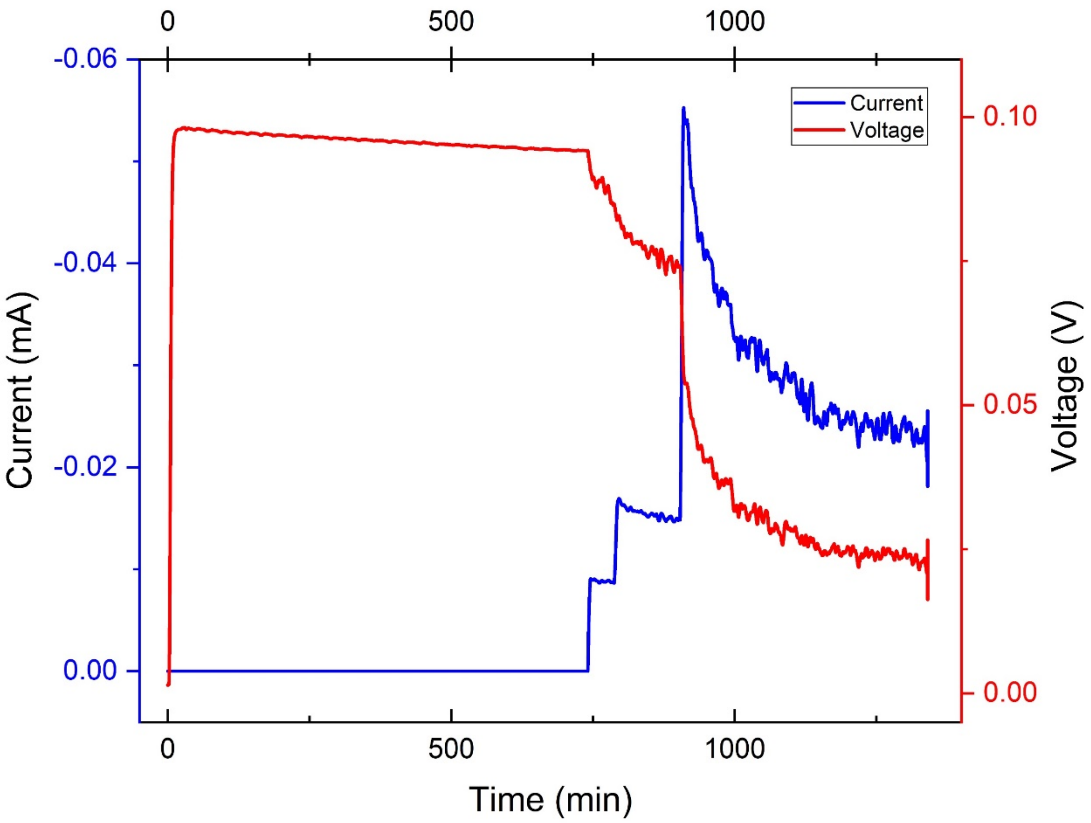

Supplementary Figure 13 - Plot of current and voltage against experimental time with the different applied resistances for the vertical cell with the gel electrolyte images shown in Figure 6. The following external load resistances were connected at the following times: 10 kΩ - 741 min, 5 kΩ - 790 min, 5 kΩ - 903 min.

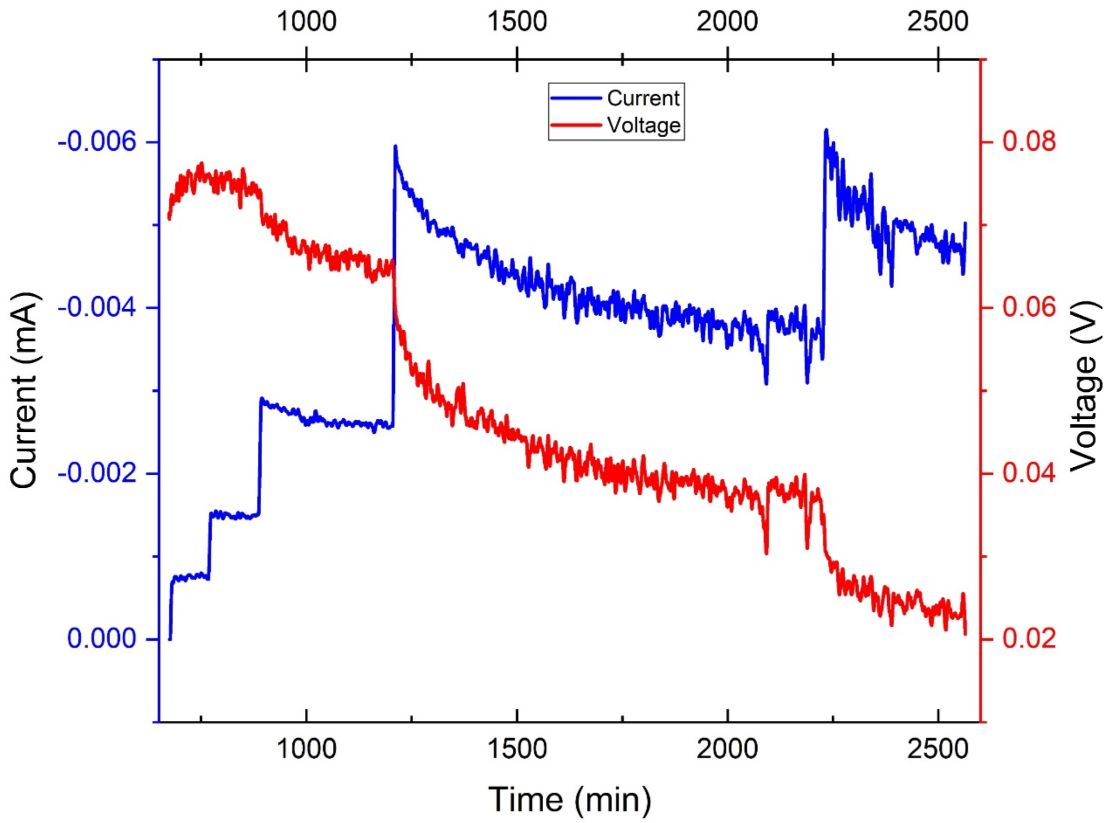

Supplementary Figure 14 - Plot of current and voltage against experimental time with the different applied resistances for the hot-above-cold cell with the gel electrolyte images shown in Figure 7a – 7g. The following external load resistances were connected at the following times: 100 kΩ - 677 min, 50 kΩ - 768 min, 25 kΩ - 887 min, 10 kΩ - 1205 min, 5 kΩ - 2226 min.

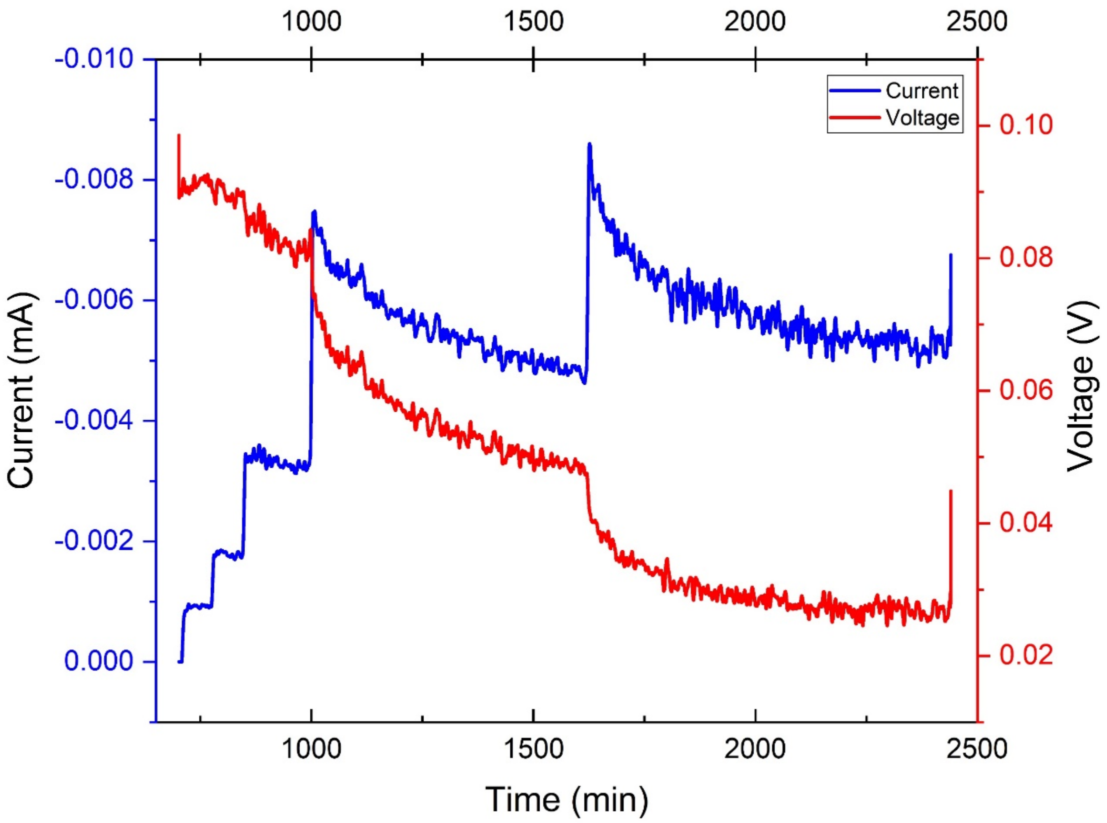

Supplementary Figure 15 - Plot of current and voltage against experimental time with the different applied resistances for the cold-above-hot cell with the gel electrolyte images shown in Figure 7h – 7n. The following external load resistances were connected at the following times: 100 kΩ - 709 min, 50 kΩ - 775 min, 25 kΩ - 846 min, 10 kΩ - 998 min, 5 kΩ - 1620 min.

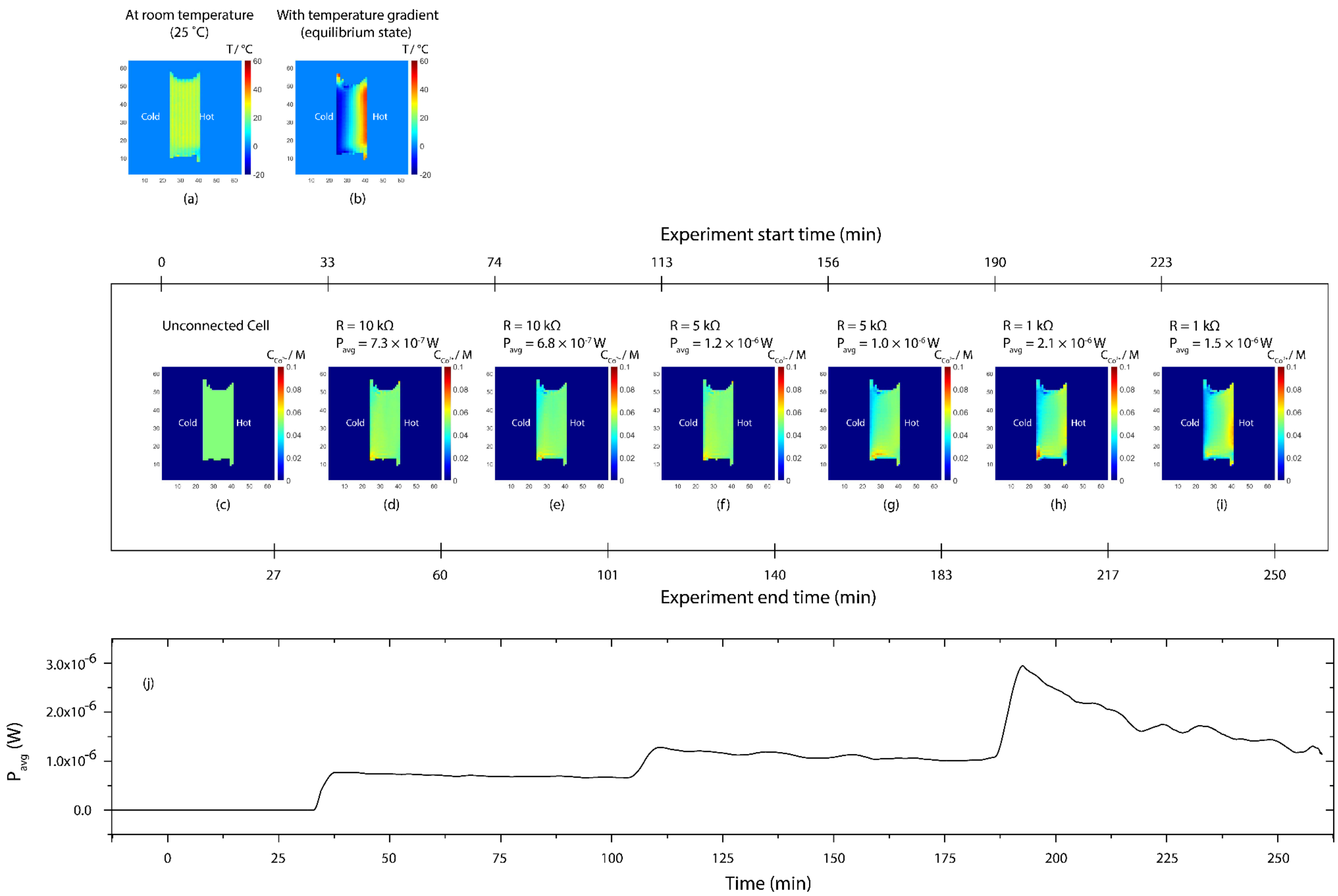

Supplementary Figure 16 – Temperature maps (a and b) and Co^{2+} concentration maps (c to i) for the vertical electrode cell under various conditions as indicated. (j) Measured cell output power as a function of time. Some minor apparent temperature variation is observed at the top and bottom of the electrolyte in (a), which we attribute to experimental artifacts.
